# Research in a time of enteroids and organoids: how the human gut model has transformed the study of enteric bacterial pathogens

**DOI:** 10.1080/19490976.2020.1795389

**Published:** 2020-08-14

**Authors:** Sridevi Ranganathan, Emily M. Smith, Jennifer D. Foulke-Abel, Eileen M. Barry

**Affiliations:** a Center for Vaccine Development and Global Health, University of Maryland School of Medicine, Baltimore, MD, USA; b Department of Medicine, Division of Gastroenterology and Hepatology, Johns Hopkins University School of Medicine, Baltimore, MD, USA

**Keywords:** Human intestinal enteroids, organoids, bacterial pathogens, enteric bacteria

## Abstract

Enteric bacterial pathogens cause significant morbidity and mortality globally. Studies in tissue culture and animal models shaped our initial understanding of these host–pathogen interactions. However, intrinsic shortcomings in these models limit their application, especially in translational applications like drug screening and vaccine development. Human intestinal enteroid and organoid models overcome some limitations of existing models and advance the study of enteric pathogens. In this review, we detail the use of human enteroids and organoids to investigate the pathogenesis of invasive bacteria *Shigella, Listeria*, and *Salmonella*, and noninvasive bacteria pathogenic *Escherichia coli, Clostridium difficile*, and *Vibrio cholerae*. We highlight how these studies confirm previously identified mechanisms and, importantly, reveal novel ones. We also discuss the challenges for model advancement, including platform engineering to integrate environmental conditions, innate immune cells and the resident microbiome, and the potential for pre-clinical testing of recently developed antimicrobial drugs and vaccines.

## Introduction

Bacterial enteric pathogens are a significant contributor to global diarrheal disease burdens.^1^ Infectious diarrhea is among the leading causes of death for children in low – and middle-income countries (LMIC).^2,3^ In the United States, elderly and immunocompromised individuals are especially susceptible to enteric infections. Enteropathogens are commonly spread through contaminated food and water or poor hygiene. The alarming increase in antibiotic resistance among enteric bacteria has increased the urgent need for therapeutic and preventative interventions.^4,5^

To study the molecular mechanisms employed by pathogenic enteric bacteria, researchers have primarily used immortalized human cell lines such as T84, HT-29, and Caco-2, human tissue explants, or small animal models including mice, rats, rabbits, and pigs. These models have greatly advanced our understanding of molecular pathogenesis by identifying host receptors for bacterial/toxin engagement, host cell surface remodeling, bacterial/toxin internalization pathways, and immune responses. However, it has also been widely recognized that these models can be inconsistent with the physiology of the human intestine.^6^ Transformed cell lines do not manifest the diversity of epithelial cell types equivalent to native intestine, and they may change genotypically with increasing passage in cell culture; as such, they do not necessarily render a complete or stable physiological response to infection.^6^ Non-transformed human intestinal explants overcome the limitations associated with cancer-derived cell lines but are short-lived and must be continuously sourced. Small animal models may lack faithful replication of human receptors, infection susceptibility, antimicrobial peptides, and innate immune responses.^7,8^

The development of human intestinal organoid mini-gut models^9,10^ presents a highly human-relevant novel platform with the potential to revolutionize the study of enteric bacterial pathogenesis as well as the evaluation of new therapeutic and preventative interventions. The central advantage of organoid models is their multicellular composition of non-transformed human cells including absorptive enterocytes, mucus-producing Goblet cells, antimicrobial peptide-producing Paneth cells, hormone-secreting enteroendocrine cells, chemosensory tuft cells, antigen-sampling microfold cells, and multipotent proliferative stem cells. Collectively, organoids retain distinct features of human intestinal epithelium, including donor genetics, segmental specification, cell polarization, nutrient and ion transport, barrier function, mucus secretion, and microbicidal peptide production.^11–13^

**Figure 1.**
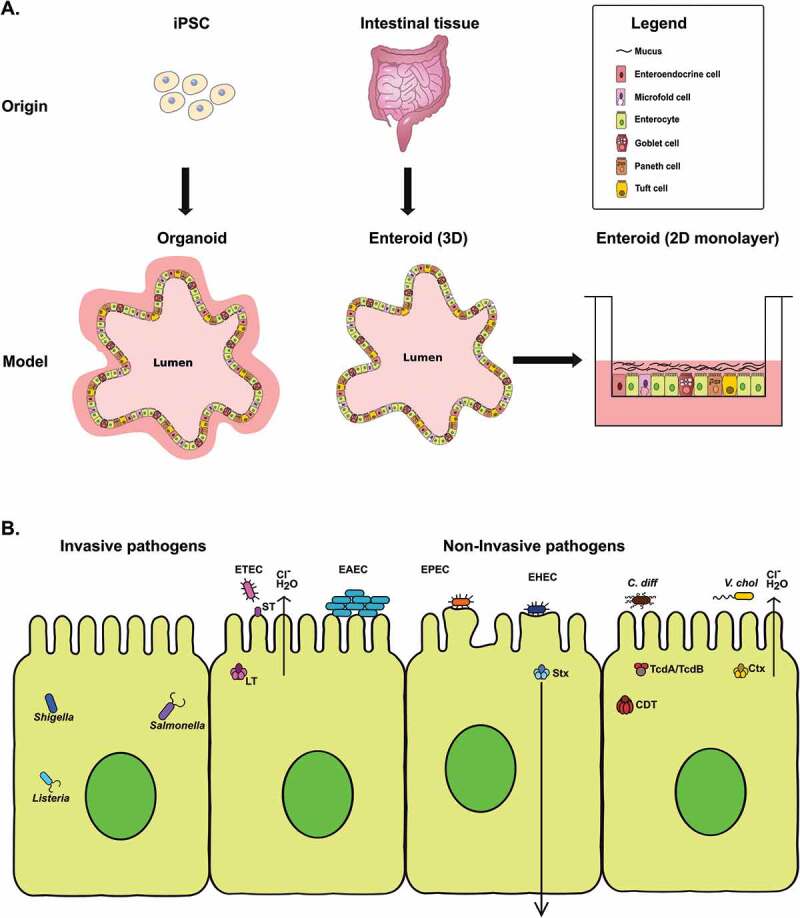
(a) Illustration of the origin and lineage composition of organoids and enteroids. Organoids are derived from induced pluripotent stem cells (iPSCs) and contain cells of epithelial and mesenchymal lineages. Enteroids are derived from intestinal tissue and contain cells of epithelial lineage only. Both enteroids and organoids contain multiple epithelial cell types. (b) Schematic diagram showing an outline of the molecular pathogenesis mechanisms of invasive and noninvasive enteric bacteria in organoids and enteroids discussed in this review. Invasive bacteria such as *Shigella, Salmonella* and *Listeria* infect and gain entry into the epithelial cells whereas noninvasive bacteria such as pathogenic *E. coli, C. difficile* and *V. cholerae* exert their effect on epithelial cells via toxins or effectors.

The term *organoid* is broadly applied to *ex vivo* cultures, and requires additional distinction as to whether it refers to an intestinal tissue-derived organoid^9^ (*enteroid*, consisting only of epithelial cells) or a human induced pluripotent stem cell (iPSC)-derived intestinal organoid^14^ (*HIO*, containing both epithelial and mesenchymal lineages) (Figure 1). Due to the maintenance of segmental specificity, enteroids derived from the large intestine are referred to as colonoids. Enteroids can be studied as three-dimensional (3D) cultures embedded in extracellular matrix (ECM) or as two-dimensional (2D) monolayers on permeable tissue culture supports. Due to tissue-like complexity, HIOs are used exclusively as 3D cultures. Enteroids can be maintained as a crypt-like population or be directed to differentiate into surface/villus-like epithelium, whereas HIOs contain both crypt and surface/villus regions simultaneously. Each model has additional unique characteristics that have been comprehensively reviewed^15,16^ and continues to evolve through manipulation of culture conditions.

In the approximately ten-year period since the introduction of mini-gut culture methods, application to the study of enteropathogens has flourished. Some of the earliest host-pathogen human organoid studies demonstrated viral entry and replication by rotavirus,^17–19^ and bacterial and parasitic^20^ infection models soon followed. In this review, we will highlight findings that include the invasive bacteria *Shigella, Listeria*, and *Salmonella*, which enter host cells and subvert the surrounding environment to facilitate intracellular replication, as well as noninvasive bacteria, including pathogenic *E. coli* (enterotoxigenic *E. coli*, enterohemorrhagic *E. coli*, enteroaggregative *E. coli*, and enteropathogenic *E.coli), Clostridium difficile*, and *Vibrio cholerae*, which associate with the cell surface, inject molecular effectors, and/or secrete toxins (Table 1). Several bacterial enteropathogen studies have utilized enteroids established from mice, but these studies will not be discussed. We summarize the technical advances, innovative methodologies, and novel discoveries of host–pathogen interaction modeling in human enteroids and HIOs to demonstrate progress in understanding human-specific disease mechanisms and the potential for preclinical testing of new therapeutics and vaccines to ultimately decrease the global burden of diarrheal diseases.

**Table 1.** Invasive and noninvasive enteric bacteria that have been studied using human organoids and enteroids and the respective research focus. *Enteroid – no mesenchyme; from duodenum, jejunum, or ileum. **Colonoid – no mesenchyme; from colon or cecum. ***HIO: human induced pluripotent stem cell derived-intestinal organoid (epithelium and mesenchyme).

Bacteria	Origin	Intestinal section	Type	Research focus	Refs
**Invasive pathogens**					
*Shigella flexneri*	Human Biopsy	Duodenum, Ileum, Colon	2D Enteroid*, 2D Colonoid**	Invasion, intracellular replication, host gene expression	21
	Human Biopsy	Duodenum, Jejunum, Ileum, Colon	2D Enteroid, 2D Colonoid	Invasion, M cells, intracellular replication, mucus changes, cytokine secretion	22
	Human Biopsy	Cecum	2D Colonoid	Testing bacteriophage therapy	23
	Human Biopsy	Colon	2D Colonoid	Adherence factor expression	24
*Listeria monocytogenes*	Human Biopsy	Ileum	3D Enteroid	Reversing enteroid polarity to facilitate apical infection	25
*Salmonella enterica* serovar Typhimurium	iPSC (dermal fibroblasts)	n/a	3D HIO***	Invasion, infection-associated transcriptional changes	26
	iPSC	n/a	3D HIO	Organoids derived from healthy iPSC and infant IBD patient; role of IL-22 in controlling infection	27
	Human Biopsy	Ileum	3D Enteroid	Reversing enteroid polarity to facilitate apical infection	25
*Salmonella enterica* serovar Typhi	Human Biopsy	Ileum	2D Enteroid	Cytoskeletal changes during infection	28
**Noninvasive pathogens**					
Enterotoxigenic *E. coli*	Human biopsy	Duodenum, jejunum	3D Enteroid	Toxin-mediated inhibition of ion transporter NHE3	29
	Human biopsy	Jejunum	2D Enteroid	Toxin-induced polarized cyclic nucleotide secretion	30
	Human biopsy	Jejunum	2D Enteroid	ST-induced pathology in Intestine Chip model	31
	Human biopsy	Jejunum, ileum	2D Enteroid	Minor adhesin-mediated adherence & cyclic nucleotide production	32
	Human biopsy	Duodenum, jejunum, colon	2D Enteroid, 2D Colonoid	Effect of macrophage co-culture on infection and epithelial cell differentiation	33
Enterohemorrhagic *E. coli*	Human biopsy	Colon	2D Colonoid	Role of mucus and SPATE EspP in infection	34
	Human biopsy	Colon	2D Colonoid	SPATE EspP-induced ion transport	35
	iPSC	n/a	3D HIO	Pathogen vs. commensal effect on epithelium; co-culture with neutrophils	36
	iPSC	n/a	3D HIO	Shiga toxin effects and mesenchymal-epithelial cross talk	37
Enteroaggregative *E. coli*	Human biopsy	Duodenum, ileum, colon	2D Enteroid, 2D Colonoid	Adherence patterns by donor and intestinal segment type	38
	Human biopsy	Colon	2D Colonoid	SPATE SepA-induced ion transport	35
Enteropathogenic *E. coli*	Human biopsy	Duodenum, jejunum, colon	2D Enteroid, 2D Colonoid	Effect of macrophage co-culture on infection and epithelial cell differentiation	33
*Clostridium difficile*	iPSC	n/a	3D HIO	Viable bacterial infection, toxin-induced pathology	39
	Human biopsy	Colon	3D Colonoid	Toxin receptor expression	40
	Human biopsy	Jejunum	2D Enteroid	Toxin-induced pathology, toxin receptor expression	41
	Human biopsy	Colon	3D Colonoid	Toxin binding to intestinal receptors	42
	iPSC	n/a	3D HIO	Role of mucus in infection	43
	iPSC	n/a	3D HIO	Dysregulation of ion transporter NHE3 and microbiota	44
	iPSC	n/a	3D HIO	Human alpha-defensin-1 and toxin neutralization	45
	iPSC	n/a	3D HIO	Bacitracin as defense against toxins	46
*Vibrio cholerae*	Human biopsy	Duodenum, jejunum	3D Enteroid	Toxin-mediated inhibition of ion transporter NHE3	29
	Human biopsy	Ileum, colon	2D Enteroid, 2D Colonoid	Toxin-induced cyclic nucleotide production	47
	Human biopsy	Rectum	3D Enteroid	Toxin inhibitors to reduce pathology	48
	Human biopsy	Rectum	3D Enteroid	Toxin inhibitors to reduce pathology	49
	Human biopsy	Jejunum	2D Enteroid	Toxin inhibitors targeting a non-canonical binding site	50
Extraintestinal pathogenic *E. coli* (ExPEC)	Human biopsy	Jejunum	2D Enteroid	Pilus-mediated adherence & invasion	51
Bacteria causing necrotizing enterocolitis (NEC)	Human biopsy, fetal	Duodenum	2D & 3D Enteroid	Immune response, maturation, & barrier function in fetal vs. adult intestine	52
	Human biopsy, fetal	Ileum	3D Enteroid	Anti-inflammatory metabolite as probiotic	53
	Human biopsy, fetal	Ileum	3D Enteroid	Human milk oligosaccharides as probiotic	54
	Human biopsy, fetal	Not specified	3D Enteroid	Cell stress and apoptosis of intestinal stem cells	55
	Human biopsy, fetal	Ileum	3D Enteroid	Impaired signaling and its role in dysfunction of intestinal regeneration	56

### Invasive enteric bacteria

Invasive pathogens penetrate the host epithelium directly through a variety of different mechanisms. These bacteria evade elimination by the host cell and use effector molecules for downstream pathogenesis. Some bacteria then spread from cell to cell while others gain access through the basolateral side of the epithelium. Invasion ultimately dysregulates intestinal cell processes and destroys the epithelium, leading to diarrheal disease.

### Shigella

*Shigella* is an invasive, Gram-negative bacterial pathogen that causes acute diarrheal disease in humans. It is associated with significant morbidity and mortality, particularly in children below 5 years of age and immunocompromised individuals.^2,57^ Current standard of care treatment includes antibiotic administration; however, the emergence of multiple antibiotic-resistant strains of *Shigella* is narrowing therapeutic options. While several vaccine candidates are in different stages of development, a licensed vaccine is not currently available.

The molecular pathogenesis mechanisms of *Shigella* have been reviewed extensively.^58–61^ *Shigella* reaches the gastrointestinal tract via ingestion of contaminated food or water or by person-to-person contact. The bacterium is able to subvert the mucus layer and reach the epithelial surface.^62^ However, *Shigella* does not infect epithelial cells efficiently via the apical surface; it exploits the microfold cells, or M cells, to transcytose the intestinal epithelium.^63–65^ Virulent *Shigella* injects over 30 effector proteins into the host cell to aid its entry.^66^ The resident macrophages at the base of the M cells phagocytose incoming bacteria, but *Shigella* escapes the phagocytic vacuole to gain access to the cytoplasm. *Shigella* then induces pyroptosis to escape the macrophage and access the basolateral surface of the epithelium.^67–69^ Intracellular replication^70,71^ and cell-to-cell spread of *Shigella*^72,73^ trigger the secretion of pro-inflammatory cytokines, including IL-8, by the epithelium,^74,75^ which recruit neutrophils to the site of the infection. Neutrophils initially exacerbate the infection by causing severe inflammation but eventually help eliminate the bacteria.^76–80^

Two independent studies using human intestinal colonoids to study *S. flexneri* pathogenesis were published in tandem in 2019.^21,22^ Both studies used colonoids derived from healthy human colon and established that human colonoids are a viable model to study *Shigella* pathogenesis. Using 2D colonoid monolayers plated on permeable membrane scaffolds, it was reaffirmed that basolateral infection by *S. flexneri* is significantly more efficient than apical infection.^21,22^ Using differentiated colonoids containing mucus-producing goblet cells, *Shigella* infection was shown to cause increased production of the major intestinal mucin glycoprotein MUC2.^22^

M cells are thought to be one of the ports of entry for *Shigella* across the epithelium.^64,81^ Complex cell culture models have been developed to induce an M cell-like phenotype in tissue culture.^82^ Using TNFα and RANKL, ileal enteroids were differentiated to include M cells^83,84^ which facilitated increased apical infection by *S. flexneri*.^22^ However, the transcytosis of bacterial cargo via the M cell was not visualized, potentially due to the long infection time periods used in the study.

Koestler *et al*. observed donor-to-donor differences in infection and intracellular replication of *S. flexneri*.^21^ In contrast to the predominantly colon-restricted pathology observed in natural cases of shigellosis in humans, *S. flexneri* was able to infect human enteroids derived from all four segments of the intestine equally.^21,22^ Differences were observed in the intracellular replication of *S. flexneri* with the lowest doubling rate in colonoids compared to enteroids derived from small intestinal segments.^22^ It was hypothesized that the dominant colonic pathology observed *in vivo* may be attributed to other factors such as peristalsis, nutrient availability, mucus layer, and microbiome that are not yet sufficiently modeled in the 2D enteroid monolayer system.

Human colonoids derived from healthy adults were used to study metabolism and immune response to infection. Koestler *et al*. used qRT-PCR to show upregulation of IL-8, TNFα, IFNβ and TNFAIP3 in colonoids apically infected with *S. flexneri*.^21^ *Shigella* infection is known to induce an amino acid starvation response and promote mTOR-mediated xenophagy in host cells.^85,86^ However, transformed cell lines with altered amino acid transport mechanisms are not suitable models to study this effect. Using qRT-PCR, Koestler *et al*. showed the upregulation of amino acid starvation response gene SLC7A5 upon *Shigella* infection and emphasized the use of colonoids with unaltered physiology to study changes in host cell metabolism upon infection.^21^ Researchers are examining the potential use of human enteroids as a preclinical model to test therapeutics against human-restricted pathogens such as *Shigella*. Llanos-Chea *et al*. used human cecum-derived colonoids to assess the efficacy of a bacteriophage against *Shigella* serotypes infection.^23^ The administration of bacteriophage significantly reduced the ability of *S. flexneri* to infect cells. Chanin *et al*. showed that exposure of *Shigella* to glucose and bile salts upregulated the expression of multiple adherence structures. The authors used colonoids to show that these adherence structures on the bacteria were important for their initial attachment to the epithelial surface.^24^

### Salmonella

*Salmonella enterica* subsp. *enterica* includes over 1400 serotypes with a wide range of pathogenic features and clinical outcomes. The most important clinical divisions are typhoidal and non-typhoidal. The four serovars Typhi, Paratyphi A, Paratyphi B and Paratyphi C constitute the group of typhoidal salmonellae that cause a serious systemic disease called typhoid or enteric fever in humans. Typhoidal *Salmonellae* are a major global health concern as they infect more than 27 million people worldwide and lead to 200,000 deaths annually.^87^ All remaining serotypes are grouped together as non-typhoidal salmonellae (NTS) which generally cause self-limited gastroenteritis, accounting for ~94 million cases and 155,000 deaths globally.^88^ Among the NTS, *Salmonella enterica* serovar Typhimurium is the most studied serotype.

Researchers studying *Salmonella* have primarily used tissue culture and small animal models to study host–pathogen interactions.^89^ *S*. Typhimurium has a broad host range including humans, livestock, small rodents, and birds; however, *S*. Typhimurium infection presents differently in each host. For instance, *S*. Typhimurium causes only localized gastroenteritis in humans but manifest as a systemic infection in mice. Typhoidal *Salmonellae* are particularly difficult to study as they are exclusively human-restricted pathogens. Humanized mice and chimpanzees have been used to study *S*. Typhi, but there is widespread recognition that a more amenable model to study *Salmonella* pathogenesis is vital for research.^89^

In order to develop an alternative model for studying *Salmonella* pathogenesis, Forbester *et al*. used HIOs to study the interaction of *S*. Typhimurium with the intestinal epithelium.^26^ In this study, HIOs were infected apically by microinjection with either *S*. Typhimurium SL1344 (wildtype strain) or a SL1344∆*invA* strain. Using a modified gentamicin protection assay, the authors showed that the *invA* mutant strain was ~30-fold less invasive than the wildtype counterpart, thus confirming previous studies that *invA* is critical for the bacterium’s ability to infect epithelial cells.^90^ The authors used a transcriptomic approach to acquire a global snapshot of the effect of *S*. Typhimurium infection in HIOs. RNA-seq of HIOs infected with *S*. Typhimurium for 3 hours showed differential regulation of several genes involved in cytokine signaling, extracellular matrix reorganization and innate immune responses.^26^ Gene expression studies showed that proinflammatory cytokines such as IL-23, IL-1β, IL-8, CXCL2 and TNFα were upregulated upon infection with *S*. Typhimurium. Cytokine measurements confirmed increases in IL-6, IL-8 and TNFα in the supernatant of infected HIOs. Using transmission electron microscopy (TEM), the authors showed that *S*. Typhimurium resided in vacuoles within the epithelial cells, confirming a hallmark of *Salmonella* infection.

In a follow-up study, Forbester *et al*. investigated the role of IL-22 in priming the antimicrobial defense of the human intestine to counteract *S*. Typhimurium infection.^27^ Organoids were generated using stem cells from a patient with infantile inflammatory bowel disease, harboring a loss-of-function mutation in the IL-10Rβ gene that encodes IL-10R2. As IL-10R2 is a component of the IL-22 receptor, patients with this mutation are non-responsive to a number of cytokines including IL-22. The authors showed that HIOs generated from healthy donor iPSCs expressed IL-22 receptors on their basolateral surface and responded to exogenous IL-22 treatment, whereas HIOs derived from the patient with a loss-of-function mutation in IL-10Rβ were non-responsive to IL-22.^27^ Healthy HIOs responded to IL-22 by upregulating the expression of genes involved in antimicrobial response such as *REG3A* and *REG1B*, mucin genes *MUC1* and *MUC4*, IFN-regulated genes *IFITM1, IFITM2* and *IFITM3*, and genes involved in maturation of epithelial oxidase complex *DUOX2, DUOXA1* and *DUOXA2*. When healthy HIOs were infected with *S*. Typhimurium concomitant with IL-22 treatment, genes involved in host defense mechanisms such as *DUOX2, LCN2* and *CXCL2* were further upregulated. Such IL-22-dependent antimicrobial responses were not observed in the HIOs derived from the IL10Rβ-mutated iPSC donor. IL-22 induced a protective phenotype in HIOs as IL-22 treated HIOs were more resistant to initial invasion by *S*. Typhimurium compared to the IL-22 non-responsive HIOs. The long-term survival of *S*. Typhimurium was also reduced in IL-22 pretreated HIOs due to calgranulin A-mediated increase in phagosome-lysosome fusion, a process that is strategically evaded by *S*. Typhimurium.^91^ This study shows the advantage of using HIOs to illustrate the effects of a cytokine receptor mutation on infection outcomes.

3D enteroids and HIOs require microinjection to introduce pathogenic material to the apical surface of epithelial cells, a tedious process that requires precise technique. Based on previous work which showed that the epithelial polarity of 3D MDCK spheroids is driven by their exposure to extracellular matrix components (ECM),^92^ Co *et al*. generated ‘apical out’ enteroids by growing 3D enteroid spheres in suspension instead of embedding them in ECM.^25^ These ‘apical out’ enteroids polarized with villin localizing on the outer edge of the spheres marking the apical surface and β-catenin along cell junctions and the inner edge marking the basolateral surface. Other than the lack of a lumen and fewer goblet cells upon differentiation, the ‘apical out’ enteroids were comparable to ECM-embedded 3D enteroids. This technical advancement of ‘apical-out’ enteroids allows for easy access to the apical surface of enteroids and was used to show the preferential apical infection by *S*. Typhimurium and basolateral infection by *L. monocytogenes*. Some hallmarks of *S*. Typhimurium infection, such as actin ruffling and infected cell extrusion, were also modeled in this system. The authors speculated that sites of cell extrusion could serve as a vulnerable site for pathogens to infect. Human enteroids serve as a valuable model to answer these early pathogenesis mechanisms.

Nickerson *et al*. primarily used an *ex vivo* human intestinal biopsy model to understand the early events of *S*. Typhi pathogenesis and confirmed key results using enteroids derived from human ileum.^28^ This study confirmed that upon *S*. Typhi infection, there was a change in cytoskeleton and microtubule dynamics of the host epithelium. The authors used inhibitors of actin polymerization and microtubule reorganization to show that invasion by *S*. Typhi decreased when cytoskeletal remodeling was inhibited.

### Noninvasive enteric bacteria

In contrast to invasive pathogens, noninvasive pathogens do not directly penetrate the host epithelium but instead attach to host intestinal epithelium to inject toxins and/or effector molecules. Intoxication of epithelial cells disrupts homeostatic signaling for a variety of cellular processes, resulting in diarrhea.

### *Enterotoxigenic* E. coli *(ETEC):*

Enterotoxigenic *Escherichia coli* (ETEC) is a leading cause of diarrheal disease in young children, particularly in less industrialized regions,^2^ and is a major causative agent of traveler’s diarrhea. ETEC adheres to the intestinal epithelium and secretes heat-stable enterotoxin (ST) and/or heat-labile enterotoxin (LT), eliciting cGMP or cAMP-mediated signaling, respectively, to dysregulate ion and water transport across the epithelium.^93^ ST is structurally homologous to the paracrine hormones guanylin and uroguanylin and activates the intestinal guanylate cyclase C (GC-C) receptor.^94^ Upon binding of ST, the cytoplasmic catalytic domain of GC-C converts GMP to cGMP, which leads to the phosphorylation and activation of the cystic fibrosis transmembrane conductance regulator (CFTR), an apical channel permeable to chloride and bicarbonate.^94^ NHE3, the brush border Na^+^/H^+^ exchanger important for intestinal absorption of sodium and water, is inhibited by ST exposure.^29^ Upon binding of LT to the receptor GM1, LT is endocytosed and activates adenylyl cyclase, leading to cAMP-induced activation of the CFTR and inhibition of NHE3.^95^

Foulke-Abel *et al*. determined that exposure to ETEC enterotoxins induced jejunal enteroid monolayers to secrete cyclic nucleotides in a polarized manner.^30^ LT exposure induced significant apical secretion of cAMP, while addition of ST significantly increased basolateral secretion of cGMP. Interestingly, intracellular cGMP accumulation was markedly restricted by phosphodiesterase PDE5-mediated degradation in enteroids and colonoids, whereas Caco-2 and T84 polarized monolayers incubated with ST yielded high amounts of both intracellular and extracellular cGMP. Toxin delivery by ETEC is known to be enhanced by several virulence factors,^96^ and this was reinforced in the observed dependence on expression of adhesins CFA/I and EtpA and the mucinase EatA by ETEC H10407 to elicit ST-induced cGMP production in enteroids.

ST was also used to probe the contribution of fluid flow and mechanical stretch to functional responses using microfluidic Intestine Chips, an innovative tool in the advancement of the enteroid model.^31^ The chip establishes an enteroid monolayer within a flexible polymer chamber containing channels that enable luminal surface fluid flow and application of culture membrane deformation to mimic peristalsis-like forces. Under continuous flow conditions, exposure of jejunal enteroid monolayers to ST increased the secreted but not intracellular cGMP concentrations and increased expression of cyclic nucleotide transporter MRP4, compared to monolayers under static conditions. Repetitive stretch did not have an additive effect on cGMP levels. When considering the aforementioned studies,^30,31^ the choice of experimental model, such as enteroids vs. immortalize cell lines or application of static vs. shear fluid forces, can give different results, and it is not yet clear which model is to be considered most reflective of native intestinal physiology or pathophysiology of ETEC infection.

As an example of using enteroids to interrogate mechanisms related to disease epidemiology, Kumar and Kuhlmann *et al*. demonstrated that the ETEC adhesin EtpA targets polysaccharides with the blood group A surface antigen.^32^ The study was driven by a meta-analysis of volunteer challenge studies that suggested ETEC caused more severe disease in individuals bearing the A type antigen. Blood group A donor jejunal and ileal enteroid monolayers were colonized more extensively by ETEC H10407 than monolayers derived from blood group B or O donors. H10407 lacking EtpA expression induced significantly decreased cyclic nucleotide levels in type A enteroids relative to wild type H10407, suggesting that enhanced colonization and toxin delivery supported by EtpA correlates with disease severity.

ETEC infection of epithelium alongside innate immune cell effectors was studied using a co-culture of enteroid or colonoid monolayers and human monocyte-derived macrophages.^33^ The enteroid monolayer and basolaterally engrafted macrophages affected each other as evidenced by the morphological changes in both cell types. Increased basal production of innate cytokines, including IL-8, IFN-γ, and IL-6, increased cell height, and transepithelial resistance (TER) across the monolayer was also observed.^33^ Following ETEC infection, cytokine production did not significantly increase; however, macrophages extended dendritic-like projections through the permeable culture membrane and paracellular space of the monolayer to contact adherent ETEC on the apical surface. Fewer ETEC bacteria were found adhered to the enteroids co-cultured with macrophages, as early as 30 min post infection, compared to enteroid-only cultures. The authors hypothesized that this decrease may be due to phagocytosis of ETEC by the macrophages. This co-culture system is the first primary human macrophage-enteroid model established to study host–pathogen interactions. Future studies aim to incorporate other immune cell types, including neutrophils, dendritic cells, and T cells, to study mechanisms of infection clearance by the immune system.

### *Enterohemorrhagic* E. coli *(EHEC):*

Enterohemorrhagic *Escherichia coli* (EHEC) causes foodborne illness worldwide, with the serotype O157:H7 associated with most *E. coli* food poisoning outbreaks in the U.S.^97^ EHEC preferentially colonizes the colon, injects virulence factors through a type 3 secretion system (T3SS) to form attaching/effacing (A/E) lesions, and remodels the actin cytoskeleton into pedestal-like structures.^98^ Shiga toxin is released into the lumen, where it binds the epithelial receptor Gb3/CD77, induces cell death through inhibition of protein synthesis, and is disseminated to other tissues.^99^ Infection is characterized by abdominal cramping and bloody diarrhea; however, severe disease can affect multiple organ systems, including the circulatory and renal systems, resulting in hemolytic uremic syndrome (HUS).^100^ HUS is characterized by clot formation, destruction of red blood cells, platelet depletion, and acute kidney failure.

The enteroid model has confirmed and extended understanding of many classical features of EHEC pathogenesis. In *et al*. used colonoid monolayers to demonstrate that EHEC preferentially adhere to differentiated monolayers that produce mucus and subsequently degrade the mucus for attachment.^34,101^ Microscopy revealed that EHEC form the characteristic A/E lesions and actin pedestals via membrane remodeling. EHEC-induced damage of intestinal cells was not limited to cells in direct contact with EHEC A/E lesions. Another hallmark of EHEC infection is the loss of microvilli; however, the molecular mechanism is poorly understood. In *et al*. showed that the serine protease autotransporter of *Enterobacteriaceae* (SPATE) EspP, produced by EHEC, specifically targeted protocadherin 24 (PCDH24), a major building block of intermicrovillar bridges, resulting in brush border effacement and remodeling. EHEC infection also induced the redistribution of tight junction protein occludin in these colonoid monolayers. While EspP has been shown to drive initial contact and membrane remodeling during EHEC infection, Tse *et al*. described a new role for EspP as an enterotoxin that alters electrolyte transport, using colonoid monolayers.^35^ EspP stimulated short circuit current in a dose-dependent and serine protease-independent mechanism in monolayers. Many bacterial enterotoxin mechanisms involve the activation of the CFTR channel.^102^ However, EspP-stimulated current was shown to be independent of CFTR in colonic monolayers.^35^ These studies suggest that EspP may be an important therapeutic target.

Karve *et al*. used 3D human organoids (HIOs) to study the pathogenic effects of Shiga toxin-producing *E. coli* O157:H7 in direct contrast to *E. coli* commensals to understand intestinal tolerance to commensals.^36^ Luminal infection with *E. coli* O157:H7 via microinjection resulted in increased bacterial replication and damage to the HIOs, characterized by extensive actin rearrangement and mucus depletion. After the induction of the bacterial SOS stress response, *E. coli* O157:H7 infection specifically induced reactive oxygen species (ROS) followed by secretion of Shiga toxin into the lumen of the HIOs. Commensal bacteria ECOR13 also replicated comparably but remained contained within the lumen and did not induce ROS production or cause cellular damage, supporting the role of the mucus layer as an innate defense barrier.

Elevated neutrophil counts are associated with the development of HUS and mortality due to EHEC infection.^103^ Karve *et al*. designed a co-culture system with the HIOs and human polymorphonuclear cells (PMNs) to compare outcomes of *E. coli* O157:H7 or commensal infection.^36^ Introducing *E. coli* O157:H7 to the media resulted in barrier function loss, PMN recruitment to the basolateral side of HIOs, and low numbers of neutrophils that transcytosed into the HIO lumen, whereas introduction of a commensal did not cause barrier function loss or recruitment of PMNs. Global transcriptional analysis using RNA-seq demonstrated that HIO infection with *E. coli* O157:H7 or the commensal strain upregulated over 70 genes, including those involved in growth factor receptor signaling and iron responses. However, infection with *E. coli* O157:H7 exclusively upregulated inflammatory responses, including IL-8 and IL-18, both key to neutrophil recruitment. This co-culture HIO model validates the recruitment of neutrophils in EHEC infection and provides a system to investigate this host–pathogen interaction at a more mechanistic level.

The early effects of Shiga toxin on human intestine are not well described. Using HIOs exposed via luminal microinjection or basolateral addition to cell culture media, Pradhan *et al*. demonstrated that purified Shiga toxin, Stx2a, disrupts epithelial barrier function after 48 hours, resulting in cell death via both necrosis and apoptosis.^37^ Transcytosis of Stx2a across the epithelium occurred while the epithelial barrier was still intact within 24 hours. Pradhan *et al*. also explored the effect of Stx2a on mesenchymal–epithelial transition in the HIOs, an established response in wound healing. Following Stx2a exposure, vimentin-positive mesenchymal cells in the HIOs expressed E-cadherin, an adhesion molecule normally restricted to epithelial cells, suggesting transition to an epithelial phenotype. Interestingly, this transition was also observed in response to other bacterial toxins, including *C. difficile* toxins TcdA and TcdB, but not in response to LPS, suggesting that this event is stimulated by general cellular damage, not by specific toxins. This is the first human cell-based study that demonstrates the role of mesenchymal cells in maintaining the integrity of the epithelial barrier upon Shiga toxin exposure.

### *Enteroaggregative* E. coli *(EAEC)*

Enteroaggregative *Escherichia coli* (EAEC) is an enteric pathogen that can cause both acute and persistent diarrhea, growth faltering, and death, particularly in children in developing countries.^104^ EAEC, named after the aggregative “stacked-brick” adherence phenotype, primarily adheres to the intestine using aggregative adherence fimbriae (AAFs), although atypical strains also exist.^105^ Infection with EAEC may manifest as a diverse array of symptoms and duration, as strains can express a varied combination of enterotoxins and virulence factors.

Rajan *et al*. used enteroid and colonoid monolayers derived from three donors to understand donor-dependent effects on EAEC adherence phenotypes.^38^ The authors observed that EAEC adherence to monolayers was independent of AafA fimbriae, whereas the aggregative phenotype was dependent on AafA, suggesting distinct mechanisms for the two behaviors. EAEC adhered to enteroid monolayers derived from duodenum and ileum in an aggregative pattern with donor-dependent differences in the phenotype. In contrast, EAEC adhered to colonoid monolayers in a mesh-like pattern and exhibited minimal adherence to jejunal monolayers. This study distinguished the aggregative phenotype of EAEC as having multiple morphologies and emphasized the contribution of the host to the disease.

EAEC SPATEs such as SepA and Pic are established effectors of colonization.^106–108^ Using colonoid monolayers, Tse *et al*. found that SepA also stimulated CFTR-dependent chloride secretion.^35^ This is in contrast to the EHEC SPATE, EspP, which stimulated CFTR-independent chloride secretion, or the EAEC SPATE, Pic, that did not affect ion transport.^35^ This study provided the first evidence of the contribution of SepA to EAEC-induced diarrhea.

### *Enteropathogenic* Escherichia coli *(EPEC)*

Enteropathogenic *Escherichia coli* (EPEC) causes diarrheal disease in humans, especially in young children. Similar to EHEC, initial attachment by EPEC is mediated by induction of the attaching and effacing (A/E) lesions that are mediated by the T3SS. Both pathotypes also affect intestinal ion transporters using various effector proteins.^109^ Previous experiments in rat jejunum suggested that EPEC extracellular serine protease C (EspC) acts as a highly potent enterotoxin and induces chloride secretion.^110^ Noel *et al*. studied interactions of EPEC with the host using a human enteroid monolayer-macrophage co-culture model.^33^ Following EPEC adherence to the enteroid monolayer, human monocyte-derived macrophages displayed phagocytic phenotypes including actin projections to interact with bacteria. EPEC infection resulted in significantly more adherent macrophages and projections across the monolayer. This is the first study of host–pathogen interaction with EPEC in a highly human-relevant model.

### *Other* Escherichia coli

Extraintestinal pathogenic *Escherichia coli* (ExPEC) include *E. coli* strains that have translocated from the intestine to distal locations, including the renal system, nervous system, and vascular system to induce urinary infections, neonatal meningitis, and septicemia, respectively.^111^ Many studies have investigated virulence factors involved in these distal infections; however, few studies have investigated the initial adherence and subsequent translocation of ExPEC from the intestine. Jejunal enteroid and Caco-2 monolayers were used to investigate the role of the adhesin FimH, a mannose-binding type 1 pilus tip protein in ExPEC.^51^ Poole *et al*. determined that wildtype and FimH-deficient ExPEC strains adhered to the monolayers at similar levels. However, subsequent invasion of the monolayers was partially dependent on FimH. These findings contrasted experiments in Caco-2 monolayers in which both adherence and invasion were FimH dependent. This study suggested that targeting FimH could attenuate ExPEC infection.

Untreated necrotizing enterocolitis (NEC) contributes to massive intestinal inflammation and necrosis with a high mortality rate in preterm infants.^112^ Previous studies suggested that early bacterial colonization of immature and underdeveloped fetal intestine contributes to inflammation. Senger *et al*. modeled NEC in infants using fetal enteroids from early (11 weeks) and late (22.5 weeks) gestational stages and compared the results with adult duodenal enteroids.^52^ Following addition of LPS or commensal *E. coli* HS to properly developed early fetal, late fetal, or adult enteroids, RNA-seq revealed that gene expression in early fetal enteroids lacked key inflammatory cytokines, including TNFα, IL-8, and IFN-γ, as well as NFkB expression. Late fetal enteroid cytokine levels were similar to adult enteroids. These data confirmed the immature immune response in infants and the subsequent negative impact upon bacterial colonization. A subsequent publication demonstrated that the molecule indole-3-lactic acid (ILA), a metabolite secreted by *Bifidobacterium longum* subspecies *infantis*, a bacterial species commonly found in breastmilk, had anti-inflammatory effects on IL-1β-treated fetal enteroids from early and late gestational stages.^53^ Treatment with ILA may serve as a probiotic for premature infants at risk for NEC. In accordance with the discovery of probiotics to combat NEC in premature infants, Wu *et al*. demonstrated that exposure to human milk oligosaccharides, also isolated from human breastmilk, increased intestinal crypt budding and the production of the mucin MUC2 in 3D human fetal ileal enteroids.^54^

NEC is characterized by the loss of intestinal stem cells by apoptosis. Previous studies demonstrated that the presence of NEC in premature human fetal enteroids was associated with increased ER stress and increased apoptosis within the intestinal crypts, compared to healthy premature and full-term fetal enteroids.^55^ The renewal of these stem cells, controlled primarily by the Wnt/β-catenin pathway, is required for gut regeneration and maintenance in response to this acute injury. Using 3D human fetal ileal enteroids isolated from infants with active NEC, Li *et al*. confirmed the previous observation of the loss of intestinal stem cells and the decreased proliferation, as compared to healthy premature fetal enteroids. Additionally, these fetal enteroids with NEC had impaired endogenous Wnt signaling at both the RNA and protein levels.^56^ The loss of stem cells and lack of regeneration in fetal enteroids with NEC was directly affected by Wnt activity as administration of Wnt rescued these phenotypes.^56^ As there is limited access to human fetal tissues and appropriate NEC models, enteroids provide a tool to investigate aspects that allow NEC to manifest in premature infants and to develop therapeutics.

### Clostridium difficile

*Clostridium difficile* is an anaerobic, spore-forming enteric bacteria that is responsible for a high burden of antibiotic-induced diarrhea and colitis in humans.^113^ Much of *C. difficile* pathogenesis has been examined using the primary toxins TcdA and TcdB, which modify cellular Rho GTPases and stimulate several negative downstream cellular effects. Hypervirulent strains also secrete *C. difficile* transferase toxin (CDT) that leads to actin – and microtubule-mediated deformations in the cell surface, increasing adherence and colonization.^114^

Leslie *et al*. performed the first experiments to demonstrate that viable obligate anaerobic *C. difficile* can persist in the lumen of HIOs for up to 12 hours and cause loss of barrier function in a toxin-dependent manner.^39^ Both TcdA and TcdB induced increased epithelial permeability over time, disrupting the cytoskeleton and cellular junctions. TcdA exerted additional effects on redistribution of E-cadherin, zonula occludens 1 (ZO-1), and actin, as compared to TcdB-injected HIOs. These findings confirmed previously identified toxin mechanisms.

Several cell surface proteins are proposed receptors for TcdB, including chondroitin-sulfate proteoglycan 4 (CSPG4), poliovirus receptor-like 3 (PVRL3), and frizzled-1/2/7 (FZD1/2/7).^115^ Schottelndreier *et al*. detected mRNA for each of these receptors in 3D colonoids from four human donors and in transformed intestinal cell lines, including Caco-2 and HT-29 cells, each with varying levels of expression.^40^ Engevik *et al*. examined cell rounding in jejunal enteroid monolayers exposed to the toxins, demonstrating TcdA to be 10-fold more effective than TcdB at inducing cytoskeletal rearrangement.^41^ Compared to immortalized cell lines, enteroids were found to express higher levels of the toxin receptors yet demonstrated less sensitivity to the toxins; this was attributed to the protective extracellular mucus layer in enteroids that is absent in other models. Mileto *et al*. demonstrated that TcdB induced pathogenic effects, particularly stem cell death, through both FZD7-dependent and – independent mechanisms in 3D colonoids, supporting evidence that receptors in addition to FZD7 are essential for toxin-induced pathology.^42^

The interaction of *C. difficile* with intestinal mucus and subsequent colonization in humans has not been thoroughly investigated. Engevik *et al*. reported that patients with *C. difficile* infection have disrupted intestinal mucus, and biopsies from infected patients exhibited almost complete loss of the loose outer MUC2 layer as assessed by confocal microscopy, exposing the firmly attached inner MUC1 layer.^43^ Loss of the MUC2 layer was recapitulated in HIOs injected with *C. difficile* alone or infected patient stool and revealed that *C. difficile* interacted with remaining MUC1. Engevik *et al*. also investigated components within the mucus layer during *C. difficile* infection. Stool from infected patients had an altered microbiota with increased levels of the phyla *Bacteroidetes* and *Proteobacteria* as well as differential expression of oligosaccharides, including GlcNAc, GalNAC, and galactose, compared to healthy donor stool. As *C. difficile* colonizes the mucus layer, it is hypothesized that the bacteria bind to these specific mucus oligosaccharides and proliferate; however, the levels of these oligosaccharides in mucus during *C. difficile* infection of HIOs were not significantly different than those in the uninfected HIOs. The complex mucus layers in the HIO model facilitated increased understanding for the role of mucus in *C. difficile* infection.

Other effects associated with *C. difficile* infection include toxin-induced dysregulation of ion channel function. Early *in vitro* studies found that *C. difficile* infection resulted in the internalization of the NHE3,^116^ and loss of NHE3 in animal models is known to yield chronic diarrhea, disrupted microbiota, and dysregulated luminal electrolytes. Engevik *et al*. demonstrated that biopsies from patients with *C. difficile* infection exhibited altered intestinal structure, decreased brush border NHE3, elevated fecal sodium, and differential microbiome composition.^44^ HIOs injected with viable *C. difficile* or infected stool supernatant had significantly decreased NHE3 mRNA and protein expression.

Many therapeutic strategies against *C. difficile* infection target TcdA and TcdB, and enteroids have provided a platform to assess preclinical efficacy. As hypoalbuminemic patients experience increased morbidity from *C. difficile* infection, di Masi *et al*. hypothesized that human serum albumin (HSA) could have a neutralizing effect on clostridial toxins.^117^ TcdA and TcdB induced epithelial damage, particularly in the crypt-like regions of HIOs; however, HIOs incubated with toxins and HSA had reduced pathology. While the mechanism is not completely understood, the authors speculate that HSA might bind to the toxins in the bloodstream and induce conformational changes, leading to toxin autoproteolysis. In another study, Fischer *et al*. investigated potential protection conferred by human alpha-defensin-1 against *C. difficile* toxins TcdA, TcdB, and CDT, finding reduced toxicity, less cytoskeletal disruption, and minimal E-cadherin mislocalization in HIOs exposed to a toxin cocktail.^45^ *In vitro* studies showed that human alpha-defensin-1 induced TcdA aggregation, but further work is required to characterize potential inhibition of downstream toxin-induced signaling.

In addition to antibiotic properties, bacitracin was previously demonstrated to protect cultured human cells from intoxication by anthrax lethal toxin and CDT.^118^ Zhu *et al*. demonstrated that bacitracin protected Caco-2 and HIOs from TcdB activity, reducing downstream glucosylation of the target protein Rac1 and preventing depolymerization of F-actin in HIOs.^46^ These experiments suggest that bacitracin, an approved drug for clinical use, has potential application to a combination therapy against *C. difficile* infection.

### Vibrio cholerae

*Vibrio cholerae* is responsible for a high burden of diarrheal morbidity and mortality globally.^119^ The primary virulence factor cholera toxin is an AB_5_ structured protein consisting of an enzymatically active A subunit that triggers ADP-ribosylation and immunogenic B subunits that mediate host cell binding.^119^ Surface receptor GM1 is a canonical binding site for the B subunit, yielding toxin internalization and cyclic nucleotide-dependent fluid hypersecretion via ion channels and transporters, such as CFTR and NHE3.^102^ Cholera toxin exposure inhibited NHE3 activity in 3D duodenal and jejunal enteroids, confirming a functional response in the human enteroid model.^29^ Cholera toxin exposure also resulted in the production of the cyclic nucleotide cAMP in 2D ileal enteroids and colonoids.^47^ Interestingly, enteroids and colonoids isolated from blood group O individuals exhibited consistently higher levels of cAMP upon cholera toxin exposure, as compared to blood group A individuals, suggesting a mechanism behind the association of severe cholera disease in blood group O individuals.^47^

In addition to the two approved vaccines for cholera, toxin inhibitors have been engineered as a therapeutic strategy. Many inhibitors are multivalent and target GM1 to prevent toxin binding.^120^ Previously, the potency of these inhibitors was evaluated by classical tests and model systems in the field, including GM1-ELISA or rabbit ileal loop assays.^119^ Zomer-van Ommen *et al*. adopted the 3D enteroid swelling assay as an alternative method to test inhibitor potency.^48^ Compared to maximal toxin-induced swelling in 3D rectal enteroids, GM1 inhibitors limited swelling by varying degrees. Strikingly, the tetravalent and pentavalent GM1 inhibitors were most potent against toxin-induced swelling. Haksar *et al*. also adopted this swelling assay using 3D rectal enteroids.^49^ The ligand meta-nitrophenyl α-galactoside (MNPG), previously shown to be a promising ligand for cholera toxin, and synthesized ligand derivatives were each conjugated to three multivalent polymers, each chosen for their potency and low cost. Some of these compounds exhibited increased inhibition of enteroid swelling upon dose-dependent cholera toxin exposure, emphasizing the potential for effective and economically practical prophylactics against cholera.^49^ An alternative strategy is to target a noncanonical site on cholera toxin with affinity for fucosylated moieties such as histo-blood group antigens. Cervin *et al*. designed polymers to target the fucosylated non-canonical binding site, finding they blocked the binding of cholera toxin to 2D jejunal enteroid monolayers; however, polymers designed to target both the canonical and non-canonical binding sites of cholera toxin were observed to block binding, intoxication, and ion secretion.^50^ Using human enteroids as a tool for inhibitor potency assays surpasses animal models in the ability to model human-specific cell surface glycosylation patterns.

Enteroid and organoid models have validated many previously identified mechanisms for noninvasive bacteria and provided insight on unrecognized pathogenic mechanisms.

## Conclusions and future perspectives

The bacterial pathogenesis studies discussed in this review, in addition to efforts using viral pathogens and parasites, demonstrate the functionality of enteroid and organoid models for infectious disease research. Many infection responses that were previously identified in cell lines or animals have been confirmed in enteroids/organoids, but some direct comparisons have revealed new model-specific phenotypes. This has been illustrated in the enhanced receptor expression yet diminished response to *C. difficile* toxins in enteroids compared to T84 and HT-29 cells,^41^ and the level and polarized secretion pattern of cGMP upon ETEC infection in enteroids compared to T84 and Caco-2 cells.^30^ The utility of enteroids/organoids is further justified in the novel mechanistic findings such as those related to intestinal segment tropism (*Shigella*, EAEC), histo-blood group antigens (ETEC), cell surface glycosylations (cholera toxin), a complex mucus layer (*Shigella*, EAEC, EHEC, *C. difficile*), and the ability to compare fetal and adult human tissue states (NEC). Each of these features is not directly mimicked in animal or immortalized cell line experiments.

While enteroids impart sophistication to epithelial tissue culture models, additional features of the GI tract such as oxygen gradients, physical forces, a diverse microbiome, vascularization, the enteric nervous system, and immune components are not yet fully represented in the model. The challenges in constructing more complex tissue culture models lay in the development of culture platforms to provide individual material support to multiple cell populations yet allow cells the freedom to interact seamlessly. Platforms should also provide access for on-demand sampling of secreted materials. The microfluidic Intestine-Chip seeded with enteroids from the small intestine^121-124^ or colon^125^ offers integrated manipulation of mechanical stretch, anaerobic compartmentalization, and endothelial interfacing. Silk scaffolding provides a highly permeable support network for enteroid monolayer communications with lamina propria.^126^ Other cell support materials such as those based on synthetic^127^ or extracellular matrix-based polymer networks^128^ remain to be implemented for enteroid co-cultures.

The engraftment of non-epithelial cell types will expand the mechanistic understanding of infection and enhance their utility for therapeutic or vaccine screening. Immune cell populations are crucial to modeling innate and adaptive responses to pathogenic antigens and provide antimicrobials to combat infection. The enteric nervous system is an understudied contributor to infectious diarrheal diseases, releasing neurotransmitters that impact epithelial ion transport and may affect other cell populations. The causal or protective role of the gut microbiome in infectious disease severity remains unclear, and interactions between competing microbes in addition to the effects of microbial metabolites on host barrier and immune function require further evaluation.

As co-culture models evolve, they present the opportunity for screening new antimicrobial compounds and vaccine candidates for toxicity and efficacy before transitioning to human clinical trials. Also, as enteroid models maintain the genetic background and segmental specificity from the original human donor, this system aligns with the advancement of personalized medicine. Patient-derived enteroids can be screened following specific therapy for early indications of treatment success.

Previous and current studies modeling bacterial pathogenesis in the human enteroid and organoid models have proven to be invaluable in the understanding the pathogenesis of both invasive and noninvasive bacteria. For some bacteria, this model has been used to study important research questions in the field; however, there are other bacteria for which this model is in early phase exploration or remains untested. These studies have provided a strong foundation to confirm pathogenic mechanisms as well as reveal novel findings and should be considered as the most relevant option to understand interactions of bacteria with the human intestine. The use of the enteroid and organoid models is expected to continue transforming the study of bacterial enteropathogens and accelerate development of critical therapeutic and preventative interventions.
